# A Case of Multisystem Inflammatory Syndrome in Children Mimicking Acute Appendicitis in a COVID-19 Pandemic Area

**DOI:** 10.7759/cureus.10722

**Published:** 2020-09-29

**Authors:** Ramon J Jackson, Hector D Chavarria, Sean M Hacking

**Affiliations:** 1 Department of Pathology and Laboratory Medicine, Donald and Barbara Zucker School of Medicine, New York, USA

**Keywords:** covid 19, mis-c, mimicker

## Abstract

The outbreak of the novel coronavirus (2019-nCoV) began in Wuhan, China and spread rapidly throughout the world. As of now, there have been numerous reports demonstrating clinical, radiological and pathological findings in adults. In children, the disease has essentially been seen as mild and self-limiting. However, more recently, children have been presenting with findings reminiscent of Kawasaki’s disease. And secondary to this, the benign nature of COVID-19 disease in children is beginning to be challenged. This phenomenon is now referred to as multisystem inflammatory syndrome in children (MIS-C). Further understanding the clinical course in MIS-C and its temporal association with coronavirus disease 2019 will be paramount for treatment and public health decision making. This correspondence describes a case of MIS-C with gastrointestinal manifestations mimicking acute appendicitis in a child presenting from a COVID-19 endemic area.

## Introduction

Until recently, the clinical course of coronavirus disease (COVID-19) has been reported as mild in children [1,2]. However, some children with COVID-19 have become critically ill and this illness is now termed multisystem inflammatory syndrome in children (MIS-C), or pediatric multisystem inflammatory syndrome (PMIS) [3]. MIS-C is a systemic hyperinflammatory syndrome, where patients present with fever and multisystem organ dysfunction. Diagnostic suspicion should be raised in the presence of unexplained persistent fever with unexplained symptomatology following exposure to COVID-19. Gastrointestinal (GI) symptoms are the most prominent in MIS-C and can present in a similar manner to many other infectious and inflammatory diseases seen in children [4]. Limited information currently exists on the clinical course of this life-threatening entity; therefore, characterizing the spectrum of MIS-C and its clinical consequences will be important for reducing both morbidity and mortality.

## Case presentation

A nine-year-old female presented to a primary care physician with a two-day history of fever and a one-day history of emesis with loss of appetite and fatigue. Her medical history was significant only for mild asthma. Physical examination was significant only for tenderness over the right lower quadrant of the abdomen, which resulted in referral to the emergency department secondary to suspicion for acute appendicitis. On arrival, the patient received testing that found her to be positive for COVID-19 immunoglobulin G antibodies with an antibody titer of 20.4, while polymerase chain reaction (PCR) testing was found to be negative for active infection. The patient also denied any previous COVID-19 symptoms or known exposure.

**Figure 1:**
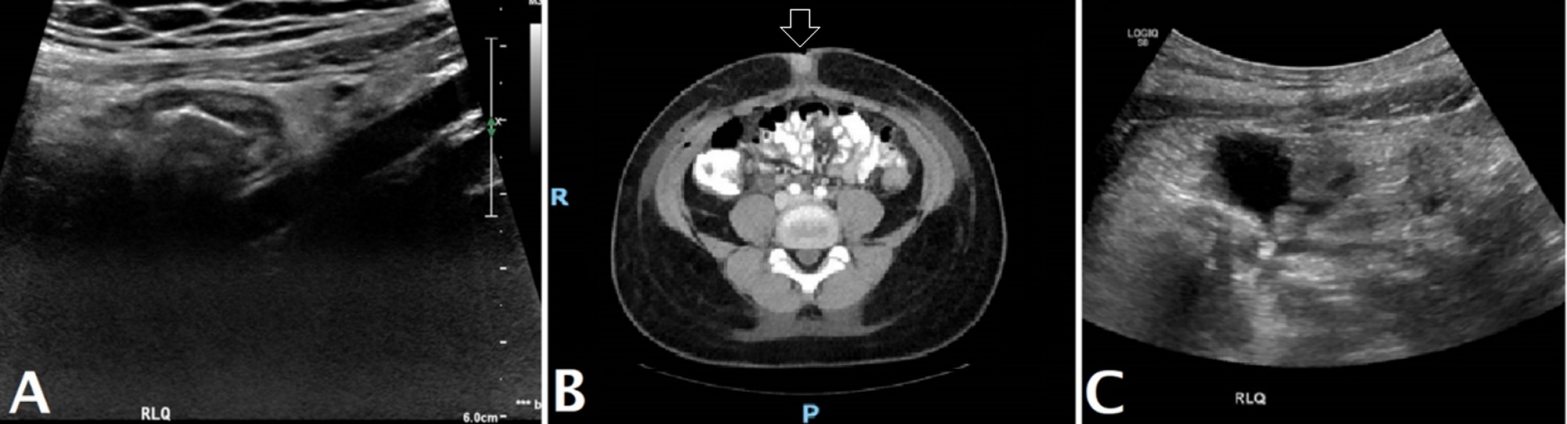
Radiology Findings A: Initial right lower quadrant abdomen ultrasound suspicious for an acute appendicitis, B: CT scan status post appendectomy and terminal ileum resection showing postsurgical changes in the right anterior abdominal wall, C: Postsurgical ultrasound showing a small amount of free fluid and inflammatory changes in the right lower quadrant.

In hospital, a right lower limited ultrasound was performed and demonstrated blind-ending tubular structure suspicious for an appendix. This structure measured approximately nine millimeter (mm) in thickness and demonstrated a "target appearance" on the transverse images. The structure was non-compressible and an appendicolith was noted at the base of the cecum. The constellation of such findings led to a clinical suspicion for acute appendicitis (Figure 1A).

She was subsequently diagnosed with acute appendicitis, admitted for an open appendectomy and began intravenous (IV) Zosyn preoperatively. The operation revealed yellow purulent peritoneal fluid and an inflamed ileum. This prompted the resection of a 3.0-cm segment of small bowel with re-anastomosis. Postoperatively, her fever and emesis resolved. However, on postop day 1 her temperature spiked to 102.1ºF. And she subsequently developed hypotension with blood pressures dipping into 80s/40s (normal, 97-112/57-71), minimally responsive to fluid resuscitation.

Postoperatively, a CT scan of the abdomen and pelvis with intravenous (IV) contrast was performed, which demonstrated mild thickening of the cecum and right colon. In the cul-de-sac, an irregularly shaped pocket of high-density fluid without enhancement was present measuring 2.5 x 0.9 x 6.1 cm, consistent with phlegmon (Figure 1B). There was also high-density fluid in the anterior prevesicular space, with mild subcutaneous air and postsurgical changes in the right anterior abdominal wall surgical site.

Sonographic evaluation of the abdomen was also performed using a high-frequency transducer, which demonstrated a small amount of free fluid and inflammatory changes in the right lower quadrant (Figure 1C).

Additionally, laboratory results for inflammatory markers including lactate dehydrogenase (LDH) and C-reactive protein (CRP) were received and found to be elevated. These results in combination with the clinical picture roused a suspicion for MIS-C, and the patient was transferred to the pediatric intensive care unit (PICU) for further treatment.

On arrival to the PICU, she was placed on maintenance fluids and standard MIS-C labs were obtained. The administration of intravenous immunoglobulin (IVIG) resulted in respiratory distress after 40 minutes; this was subsequently terminated. And the patient was given bilevel positive airway pressure (BiPAP), IV methylprednisolone 1 milligram (mg)/kilogram (kg) and aspirin 81 mg. This treatment regimen resulted in abrupt resolution of the immediate symptoms. Further examination demonstrated a normal electrocardiogram (EKG) and echocardiogram. She remained hemodynamically stable throughout the remainder of the PICU course and was weaned to room air and per os (PO) prednisolone 30 mg. MIS-C labs were trended, with progressive improvement following completion of five days of Zosyn and she was downgraded to a floor bed (Table 1).

**Table 1:** Overview of Laboratory Values WBC, White Blood Cell; LDH, Lactate Dehydrogenase; CRP, C-Reactive Protein; IgG, Immunoglobulin G; PCR, Polymerase Chain Reaction.

Variables	Day 1	Day 2	Day 3	Day 4	Day 5	Day 6	Normal range
WBC (K/µL)	7.0	6.2	8.9	8.4	12.0	9.3	4.5-13.5
LDH (U/L)	293	-	-	214	-	-	135-225
Ferritin (ng/mL)	916	-	870	-	-	-	30-400
CRP (mg/L)	19.2	177.2	228.4	207.4	94	52.2	<5
COVID IgG	20.4	-	-	-	-	-	<0.99
COVID PCR	Negative	Negative	-	-	-	-	NA

The patient was transferred safely to the floor after four days in the PICU. Inflammatory markers continued to downtrend toward normal ranges over the next two days at which time she had completed seven total days of Zosyn. A regular diet was resumed and tolerated well. She continued to be afebrile and had adequate stooling and urine output. The patient’s symptoms had completely resolved at the time of discharge on the eighth day.

Gross examination of the resected specimen revealed a 6.5-centimeter (cm) segment of unoriented small bowel and appendix with a diameter of 4.5 cm. The serosa was red-purple in color with a congested and smooth appearance. On the mucosal surface, multiple pink-tan nodular excrescences were present ranging from 0.1 to 2.5 cm in greatest dimension.

**Figure 2:**
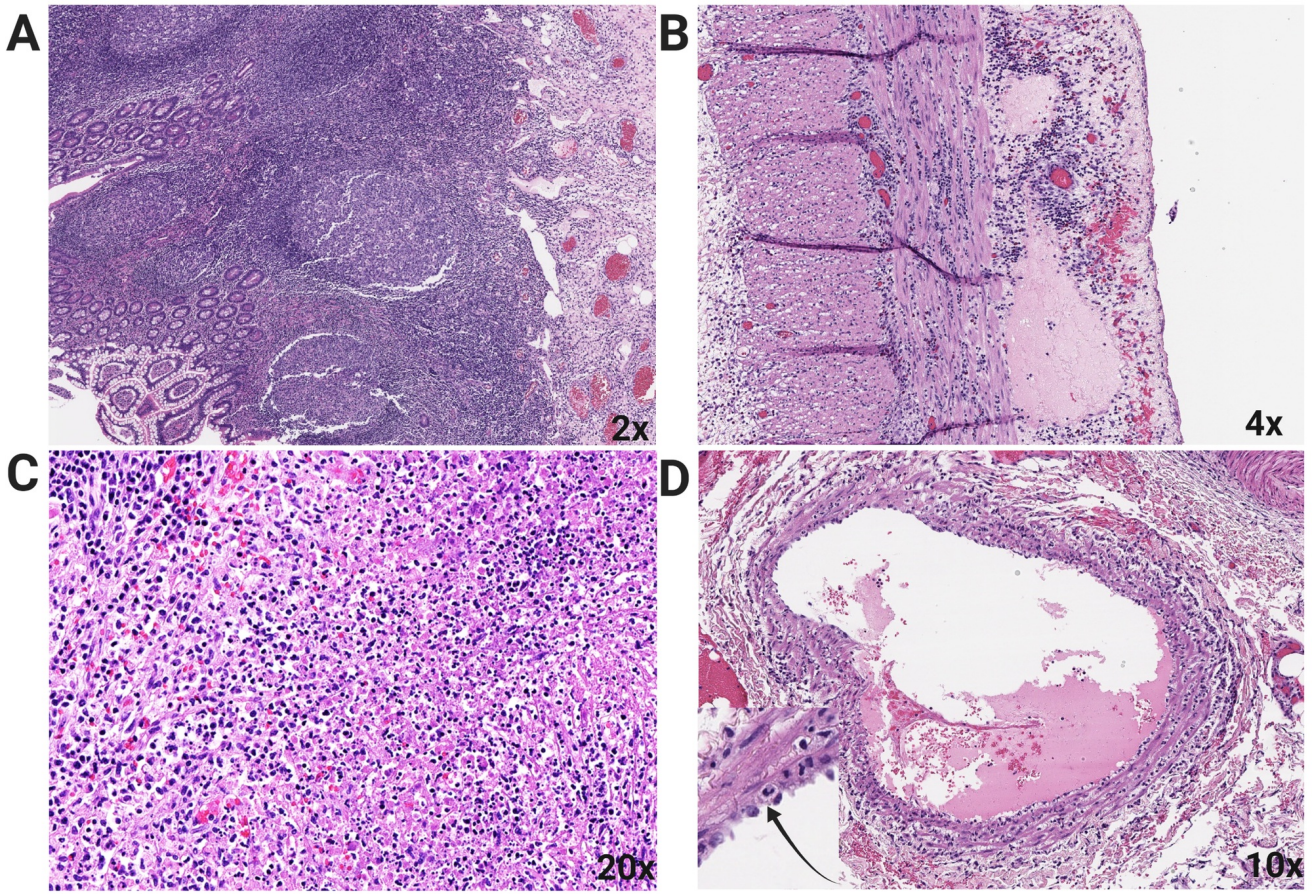
Pathology Findings on Hematoxylin and Eosin Stained Tissues A: Peyer's patches showing hyperplastic lymphoid tissue, B: Small bowel and appendix with extensive subserosal edema, C: Mesenteric lymph node with necrotizing lymphadenitis, D: Acute vasculitis of mesenteric arteries and veins (inset with arrow identifying neutrophil).

Histopathology revealed patchy mucosal inflammation, including mild acute cryptitis, occasional lamina propria neutrophils, transmural chronic inflammation with lymphocytes and eosinophils. Peyer's patches were identified showing hyperplastic lymphoid tissue, while the mucosa showed no evidence of ulceration (Figure 2A). In sections of small bowel and appendix, extensive subserosal edema was identified (Figure 2B). A mesenteric lymph node demonstrated necrotizing lymphadenitis with prominent necrotic foci (Figure 2C). In the mesentery, acute vasculitis was identified in several arteries and veins with prominent inflammatory infiltrates in adjacent mesenteric adipose tissue (Figure 2D). No evidence of granulomas or viral cytopathic effect was identified.

## Discussion

Multisystem inflammatory syndrome in children or MIS-C represents an uncommon albeit serious disease manifestation [5]. It is novel and appears to be linked to severe acute respiratory syndrome coronavirus 2 (SARS-CoV-2), the virus responsible for COVID-19 [3].

Clinical findings mirror that of Kawasaki's disease, with fever and inflammation seen in many organs including heart, lungs, kidneys, liver, skin, eyes, brain and the GI tracts [3,6]. As of now, GI involvement appears to be the predominate pattern in MIS-C and seen in 92% of patients [7]. These GI symptoms have the potential to mimic infectious GI etiologies, as well as inflammatory bowel diseases. In this case, the clinical findings led to a clinical suspicion of acute appendicitis with subsequent surgical intervention.

Current treatment recommendations for MIS-C are not concrete. And for our patient, it is unknown whether medical treatment would have been beneficial or prevented the need for surgery. In future, characterizing the epidemiology, disease spectrum, treatment modalities and prognosis of MIS-C will be key to reducing morbidity and mortality. 

Pathologic examination of the resection specimen was important; the appendix and segment of small bowel revealed findings that were discordant with the diagnosis of acute appendicitis. The anomalies found in the mesentery, including necrotizing lymphadenitis and vasculitis, were more consistent with a systemic hyperinflammatory disorder. Given that this child was presenting from an area with high COVID-19 infection rates and our patient had a positive screen for COVID-19 antibodies, the findings were most consistent with MIS-C.

Clinical correlation was important as the histology findings were not entirely specific. For example, Kikuchi’s disease commonly presents with fever in children and findings of necrotizing lymphadenitis with vasculitis are typical [8]. However, Kikuchi’s disease almost never extends into perinodal tissue as seen in our case [9]. It tends to be more long standing and has no known association with COVID-19. 

Another differential in this case includes systemic lupus erythematous lymphadenitis. Although necrotizing lymphadenitis and vasculitis can be seen, a lack of other systemic manifestations, the patient age and negative lupus serology favors against this diagnosis [10]. 

It is important to also mention necrotizing granulomatous lesions. Etiologies include both typical and atypical mycobacteria as well as fungal organisms. These lesions contain prominent granulomas; however, a finding is not seen in our case [11]. The presence of granulomas and vasculitis would favor Wegener's granulomatosis (WG), which would need to be excluded from infectious etiologies by Gömöri methenamine silver (GMS) and acid-fast bacilli (AFB) special stains [12].

Regarding Kawasaki’s disease, digestive tract involvement can be seen in 20%-35% of cases [13-15]. GI symptoms of Kawasaki’s disease can complicate clinical recognition, lead to unnecessary surgical interventions and cause therapeutic delay in a similar manner seen in our case [13,16]. Abdominal manifestations in Kawasaki’s disease have also been found to predispose to severe coronary involvement, probably secondary to a more severe and diffuse vasculitis [13]. Such associations, if present, are yet to be discovered in MIS-C. Regarding risk, one study in France demonstrated MIS-C to be more common among children of African ancestry; this was also the case for our patient [17].

Another anomaly, published in The Journal of the American Medical Associations most recent report: “Multisystem Inflammatory Syndrome Related to COVID-19 in Previously Healthy Children and Adolescents in New York City”, suggests that MIS-C cases seem to lack elevated tumor necrosis factor-alpha (TNF-α) and interleukin-13 (IL-13), normally found in acute pulmonary COVID-19 infections [5]. Further associations are likely still yet to be discovered as this pandemic progresses; however, the finding of leukopenia also seems to be prevalent [18]. And in our case, white blood cell (WBC) values were within normal limits, while other inflammatory markers including LDH and CRP protein were significantly elevated. This highlights the need for more extensive laboratory testing, where results need to be correlated with different disease differentials in mind. 

It is important to discuss that our patient denied any previous symptoms or known exposure to COVID-19 and presented with positive antibodies despite negative PRC testing. This suggests that there may be a delay in the COVID-19 immune response in MIS-C and that disease sequelae may occur long after infection with COVID-19. However, such determinations, based off one case report, would be entirely premature.

## Conclusions

MIS-C should be considered in patients with prominent GI symptoms and a history of recent SARS-CoV-2 exposure or infection. Although not definitive, MIS-C could differ from many other conditions by multiorgan involvement and extremely high inflammatory markers without elevations in WBCs, TNF-α and IL-13.

Long-term follow-up analyzing potential sequelae and organ dysfunction will be important for defining the real severity of disease from COVID-19 and MIS-C. And while every child presenting with MIS-C will have a potentially different clinical course, both the diagnosis of MIS-C and subsequent therapeutics strategies should be made with input from multiple consultant specialists.
